# Strength and water retention behavior of loess stabilized with guar gum and fiber under dry and wet cycles

**DOI:** 10.1038/s41598-025-96390-z

**Published:** 2025-04-03

**Authors:** Xinxin Du, Hao Tian, Xin Kang, Zengchun Sun, Xiaoxiao Zhao, Yucong Ren

**Affiliations:** 1 College of Mechanical and Electronic Engineering, Northwest A&F University, Yangling, 712100 Shaanxi China; 2 College of Water Resources and Architectural Engineering, Northwest A&F University, Yangling, 712100 Shaanxi China; 3 Shaanxi Provincial Land Engineering Construction Group Co., Ltd., Xi’an, 710075 Shaanxi China; 4 Key Laboratory of Degraded and Unused Land Consolidation Engineering of the Ministry of Natural Resources, Xi’an, 710075 Shaanxi China

**Keywords:** Loess stabilization, Guar gum, Dry–wet degradation, Water stability, Strength, Soil water characteristic curve, Civil engineering, Biomaterials

## Abstract

To improve the mechanical and water-retention behavior of loess and reduce the erosion failure caused by dry–wet cycles, the applicability of guar gum (GG) biopolymer and basalt fiber in the solidification of loess is investigated. The addition of GG can enhance the compressive strength and disintegration resistance of loess. When the GG content is 0.5%, 1.0%, and 2.0%, the compressive strength of stabilized loess increased by 30.15%, 67.85%, and 124.8%, respectively. The shear strength of GG–fiber stabilized loess is obviously higher than that of specimens without GG, and the higher the GG content, the stronger the shear resistance. The dry–wet cycles have a significant degradation effect on untreated and GG–fiber stabilized loess. After 8 dry–wet cycles, the cohesion and internal friction angle of the specimen containing 2.0% GG decreased by 45.90% and 10.74%, respectively. As the GG content increases, the water-retention capacity of stabilized is enhanced, but the dry–wet cycles have a significant deterioration effect. Furthermore, the soil water characteristic curves prediction model for GG–fiber stabilized loess is established by considering the effect of dry–wet cycles and GG content, and the prediction results are basically consistent with the measured data (*R*^2^ = 0.92). This study confirmed the feasibility of applying guar gum and basalt fiber to improve soil strength, water stability, and water-retention capacity, and provided a basis for engineering construction and soil erosion control in the loess area.

## Introduction

Loess is a kind of aeolian sediment and widely distributed in the northwest of China, which exhibits poor engineering properties, such as large pore structure, low strength, high compressibility and water sensitivity^1^. In addition, the long-term drying–wetting or heating–cooling effects lead to the accumulation and expansion of cracks and deformation in the soil, which induces engineering issues and geological disasters^2–5^. Traditionally, soil improvement has been achieved through various methods. For instance, the use of alkaline materials, such as lime, in soil stabilization results in the generation of CO_2_ and other industrial waste gases, contributing to environmental pollution. On the other hand, the addition of organic materials can enhance the soil’s water retention capacity and resistance to erosion. However, this approach may lead to the risk of biological accumulation. Therefore, to meet the engineering construction and disaster prevention in loess area, the solidification and improvement of loess has been the research hotspot of geotechnical engineering. With the development of the concept of environmental protection, many scholars try to use new biological technologies to replace traditional inorganic materials for soil improvement, such as Portland cement. Among them, microbial or urease-induced calcium carbonate precipitation (MICP/EICP)^6–8^ and biopolymer-based soil treatment (BPST)^9–12^ technologies have been widely concerned.

MICP and EICP are a new environmentally friendly treatment technologies for rock and soils, which use bio-induced carbonate to bond loose soil particles together, achieving the purpose of soil reinforcement^13,14^. The difference between the two methods is that MICP utilizes active microorganisms capable of secreting urease, such as *Sporosarcina pasteurii*, while EICP requires urease extracted from microorganisms or plants beforehand^15–17^. However, it is difficult to predict and design the production rate and precipitation behavior of calcium carbonate in the above methods in practical engineering^18^. In contrast, BPST technology uses extracellular polysaccharides produced by microorganisms or algae to mix with water and soils to improve the physical and mechanical properties of soils. BPST technology is not only easy to obtain and inexpensive, but also can be naturally decomposed into monosaccharides and water by microorganisms and enzymes in the soil, with almost no impact on the environment. Moreover, biopolymers are widely used in geotechnical engineering due to their high efficiency and stability in soil reinforcement^19–21^.

There are various types of biopolymers, including lignin, xanthan gum, guar gum, carrageenan, gellan gum, sodium alginate, etc.^22^. Due to differences in molecular structure and molecular weight, different types of biopolymers have varying strengthening effects on soils. For example, Chang et al.^9^ observed that xanthan gum had the greatest efficiency solidification effect on well-graded fine-grained soils and was affected by the degree of hydration of the soil. Jia et al.^23^ investigated the shear strength, disintegration resistance, permeability resistance, erosion resistance and vegetation growth of loess with different guar gum contents, and pointed out that guar gum-stabilized loess has excellent erosion resistance and water retention ability. Zhang et al.^24^ studied the water-retention behavior and microstructure of sand solidified by xanthan gum and gellan gum. Chen et al.^25^ pointed out that the strength of xanthan gum stabilized soil decreased obvious in the first four wetting–drying cycles, and then tended to stabilize. Liu et al.^26^ found that bio-based materials (calcium alginate and xanthan gum) can remarkably improve the strength, disintegration resistance and water stability of loess. Ayeldeen et al.^27^, Chen et al.^28^, and Bozyigit et al.^29^ compared and analyzed the strengthening effects of several biopolymers on soils and pointed out that guar gum was more effective than xanthan gum in strengthening strength, improving permeability and enhancing water retention capacity.

However, the application of biopolymers in soil stabilization and solidification still has some defects, such as unsatisfactory shear and tensile strength, and severe strength loss after water exposure^30^. Thus, some other materials combined with biopolymers are proposed to improve the engineering properties of solidified soils. Fiber reinforcement, the interlocking effect between fiber and soil particles, can effectively enhance the strength and toughness of soil^31,32^. Simultaneously, it can also inhibit the development of cracks, and improve soils durability and erosion resistance. Feng et al.^30^ analyzed the mechanical performance of dredged soil solidified with different xanthan gum content and jute fiber (content and length), indicating that the cementation of xanthan gum and the network structure formed between fiber and soil particles play an important role. Liu et al.^33^ explored the role of anionic polysaccharide and palm fiber on sand–clay mixtures in terms of cracking and erosion resistance.

In this context, this paper intends to investigate the impacts of guar gum (GG) and basalt fiber on the strength and water-retention behavior of loess under drying–wetting effects. Firstly, the influence of GG and fiber on the strengthening effect of loess is studied through unconfined compressive strength (UCS) test to determine the optimal content. Then, the water stability, shear strength and water-retention behaviors of stabilized loess under drying–wetting effects are explored, and a modified van Genuchten (VG) model is established to reflect the combined effect of guar gum and fiber. Moreover, the microstructure of stabilized loess is analyzed to reveal the underlying mechanisms of the two reinforcement materials.

## Materials and methods

### Materials

**Fig. 1:**
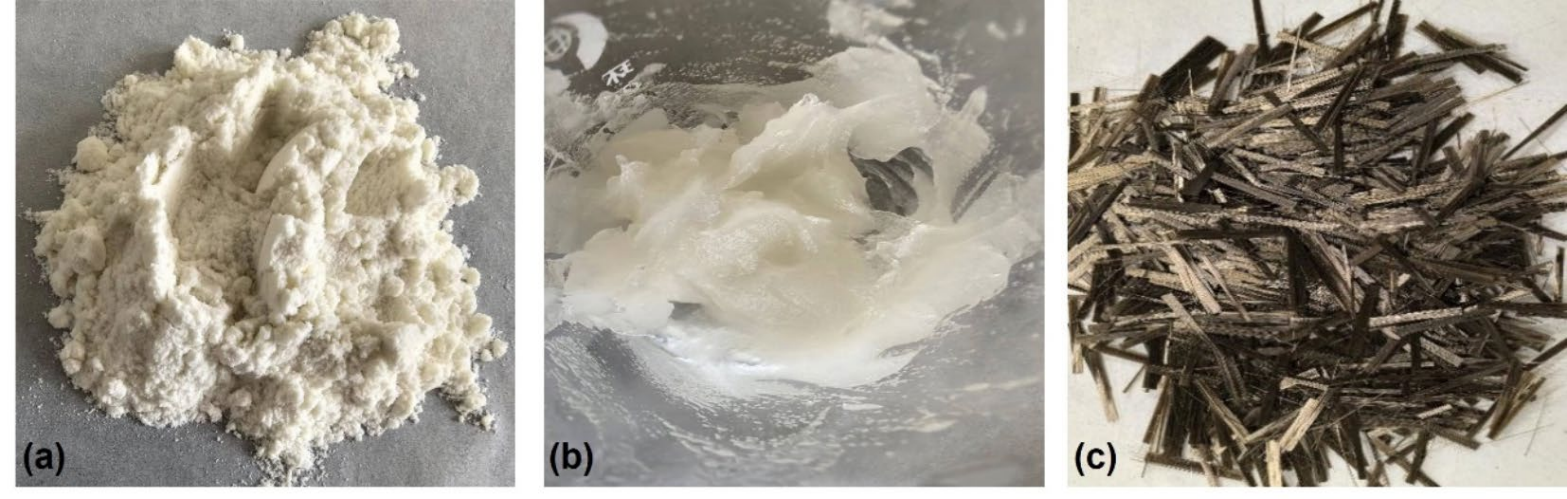
Guar gum (powder and hydrated) and basalt fiber.

The loess utilized in this study was collected from a foundation pit in Xi’an, China, with a depth of 3–4 m. The retrieved loess was naturally air-dried and pulverized, then passed through a 2 mm sieve. According to ASTM standards^34,35^, the basic properties of loess are summarized in Table 1. Guar gum is a polysaccharide-based biopolymer extracted from guar beans, which has excellent cold-water solubility, pH stability and biocompatibility^11,23^. The guar gum employed in the experimental procedure was manufactured by Beijing Guairun Technology Co., Ltd, which appears as a white free-flowing powder in the dry state and presents a highly viscous colloidal solution at low water content, as shown in Fig. 1a and b. It is often used as an emulsion stabilizer and thickener and is widely used in food, pharmaceutical, textile, printing, and other industries. Compared with other types of fibers, basalt fibers are promising for engineering applications in enhancing the mechanical properties of soils due to their excellent properties^31,36,37^, as shown in Fig. 1c.

**Table 1:** Basic properties of loess and basalt fibers.

Property	Symbol	Value	Unit
Loess			
Specific gravity	*G*_*s*_	2.70	–
Plastic limit	*PL*	17.80	%
Liquid limit	*LL*	30.50	%
Maximum dry density	$\rho_{d{\max }}$	1.63	g/cm^3^
Optimum moisture content	$w_{op}$	17.50	%
Basalt fiber			
Density	$\rho_{bf}$	2.65	g/cm^3^
Diameter	$D_{bf}$	17	mm
Length	$L_{bf}$	12	mm
Tensile strength	$T_{bf}$	1256	MPa
Elongation to fracture	$E_{bf}$	3.01	%

### Sample preparation

The specimens were prepared by dry mixing method, firstly, the sieved loess and guar gum powder were mixed thoroughly and stirred, and the GG-loess mixture was divided into three layers to add a quantitative amount of water, and the total amount of water added should meet the optimal water content (17.5%). Subsequently, the mixed soil is sealed and preserved with plastic wrap for 24 h. Basalt fibers are added and stirred until the fibers are uniformly distributed in the mixtures. After the specimen is made using the static compression method, it is wrapped in double-layer plastic wrap and placed in a curing box for 7 days at a temperature of 20 °C and a relative humidity of 95%.

**Fig. 2:**
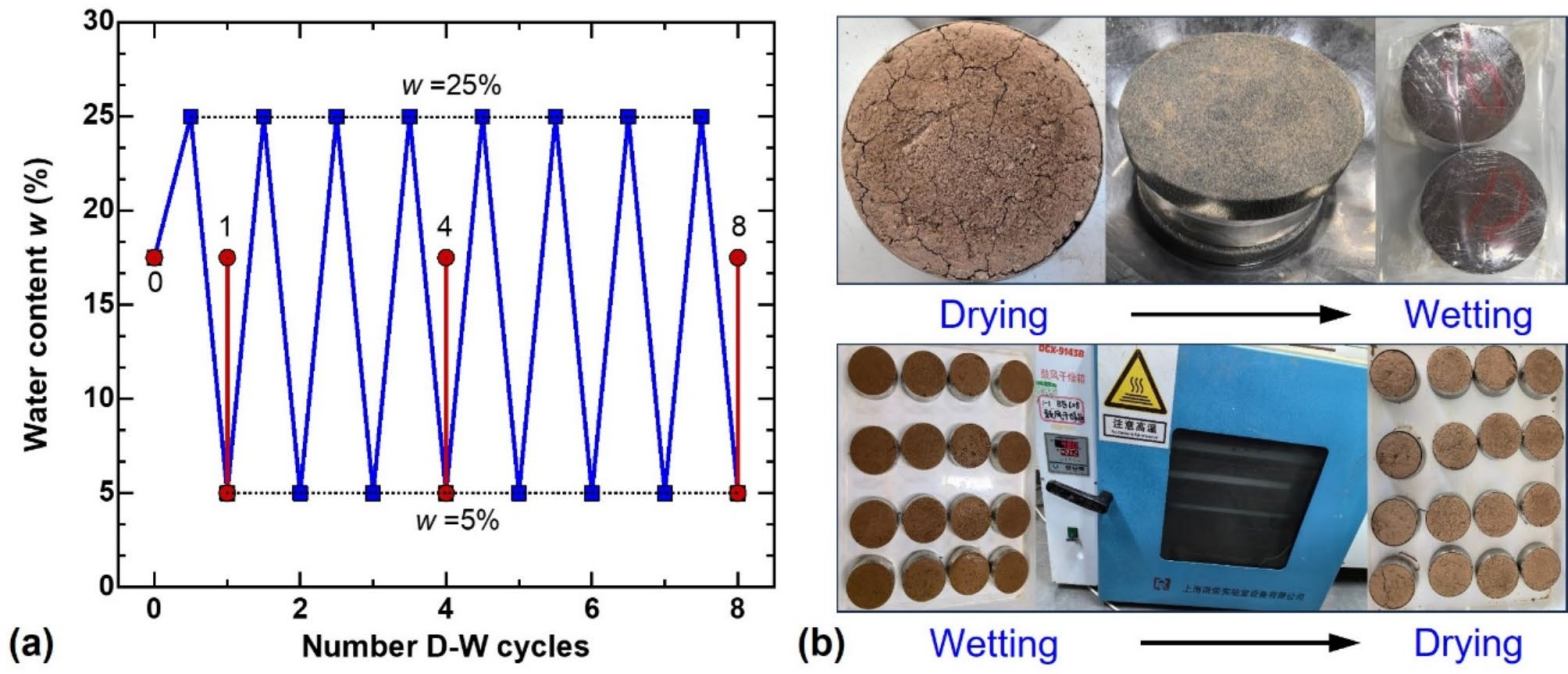
(**a**) Drying–wetting path; (**b**) wetting and drying process of specimens.

The specimens were treated by oven-dried and water-film transfer methods to analyze the strength deterioration and water-retention characteristics of guar gum–fiber stabilized loess under drying–wetting cycles. The water content of the specimens during the dry–wet cycles is controlled to be 5–25%, and the cycle number is set to be *N* = 0, 4, 8, and 12, respectively (Fig. 2a). The detailed process is as follows: (1) wetting stage: porous stone and filter paper are placed at both ends of the specimen, and water is slowly dripped onto the upper porous stone. The target water content (25%) is confirmed by repeatedly measuring the mass of the specimen. To ensure the internal moisture equilibrium, wrap the specimen with plastic wrap and place it in a curing box for 12 h; (2) drying stage: the specimens that have completed the wetting state are placed in an oven and dewatered at a temperature of 50 °C for 12 h. The change in moisture content of the specimen is recorded, and the specimen is removed when the moisture content decreases to 5%, indicating the end of one cycle, as illustrated in Fig. 2b.

### Test procedure

Considering the content of basalt fiber (FR = 0.2%, 0.4%, 0.6%, and 0.8%) and guar gum (GG = 0.0%, 0.5%, 1.0%, and 2.0%), cylindrical specimen with diameter of 50 mm and height of 100 mm are prepared. The UCS test is conducted with a strain-controlled unconfined pressure instrument to determine the optimal content of basalt fiber and guar gum. The axial strain rate is controlled at 1.5 mm/min, and the stress–strain value is recorded.

To explore the water-stability of GG–fiber stabilized loess, water immersion tests are conducted using transparent glass containers. The dimensions of the specimens were the same as those of the unconfined compression test. The specimens were first slowly put into the center of the container, and pure water was slowly poured along the wall of the container until the surface of the water exceeded the specimens, and record the state of the specimen after soaking for 30 s, 10 min, 1 h, and 24 h.

Shear strength is an important index to evaluate the effect of biopolymer on soil reinforcement. A strain-controlled direct shear instrument is used to perform a rapid shear test on the cylinder specimen of 61.8 × 20 mm, and the shear rate is set at 0.8 mm/min. The vertical stresses applied are 50 kPa, 100 kPa, 200 kPa, and 400 kPa respectively.

The soil water characteristic curve can reflect the distribution and variation of soil moisture during water absorption and drainage process, and is an important means to investigate the relationship between suction and water content. The matrix suction range of the pressure plate device is 0–1500 kPa, and cylindrical specimen with diameter of 53 mm and height of 10 mm are prepared. The pressure levels are set to 15, 20, 35, 50, 100, 160, 250, 380, 520, 825, and 1300 kPa. Each level of pressure equilibrium time is 24 h, when the pressure is higher than 520 kPa the time is extended until the specimen no longer exits water and reaches the suction equilibrium state.

## Results and analysis

### Unconfined compressive strength

**Fig. 3:**
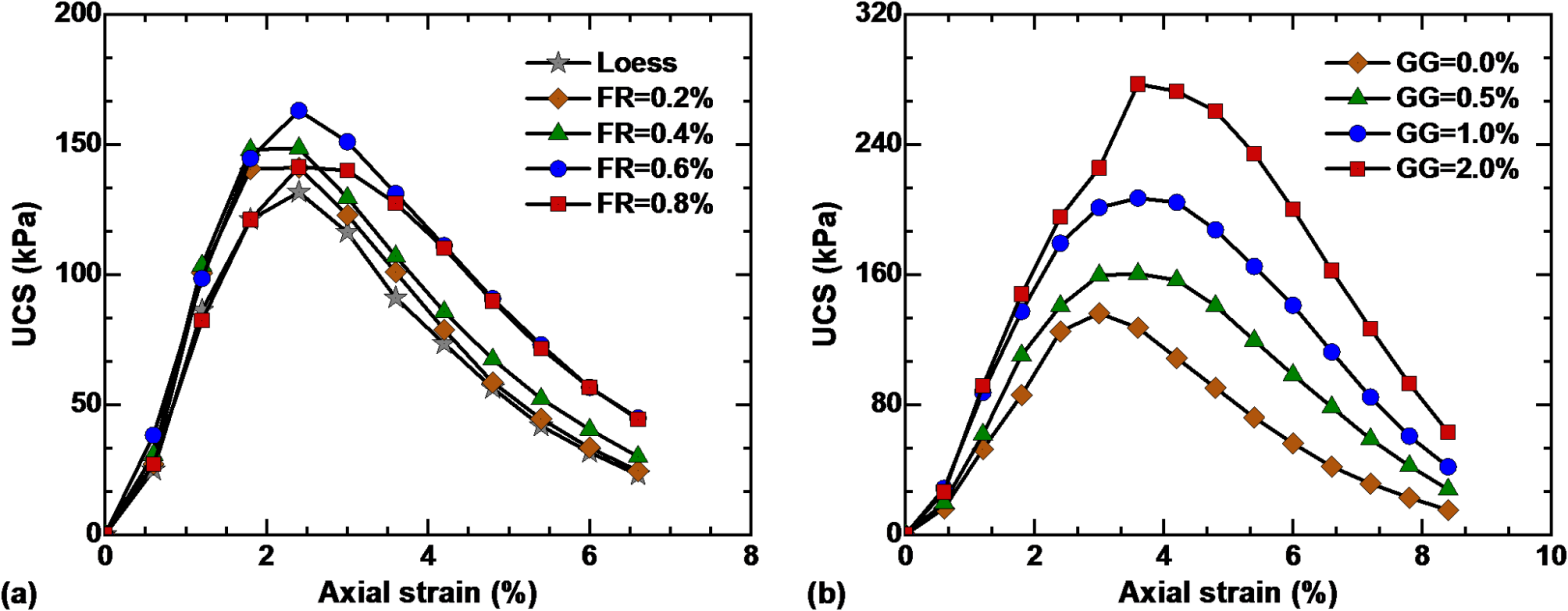
UCS of stabilized loess: (**a**) effect of basalt fiber; (**b**) effect of guar gum.

Figure 3a shows the strain–strain curves of untreated and basalt fiber-reinforced loess. The addition of fiber can increase the compressive strength of loess, and the maximum strength is reached when the fiber content reaches 0.6%, which is consistent with the conclusion obtained by Wu et al.^37^. The fibers form a network support in the soil and restrict each other under the action of load, which greatly limits the movement of soil particles and improves the compressive strength. When the fiber content exceeds the threshold value, the agglomeration phenomenon of fibers becomes more pronounced, leading to the destruction of the original structure of soil particles and a decrease in compressive strength. Therefore, the optimal fiber content of 0.6% is adopted in the subsequent experiments. The influence of GG content on the compressive strength of solidified loess is presented in Fig. 3b. The hydrogel produced by the hydration of guar gum has strong viscosity, and the compressive strength increases obvious with increasing GG content within this range. When the GG content is 0.5%, 1.0%, and 2.0%, the strength of solidified loess increased by 30.15%, 67.85%, and 124.8%, respectively.

### Water stability

**Fig. 4:**
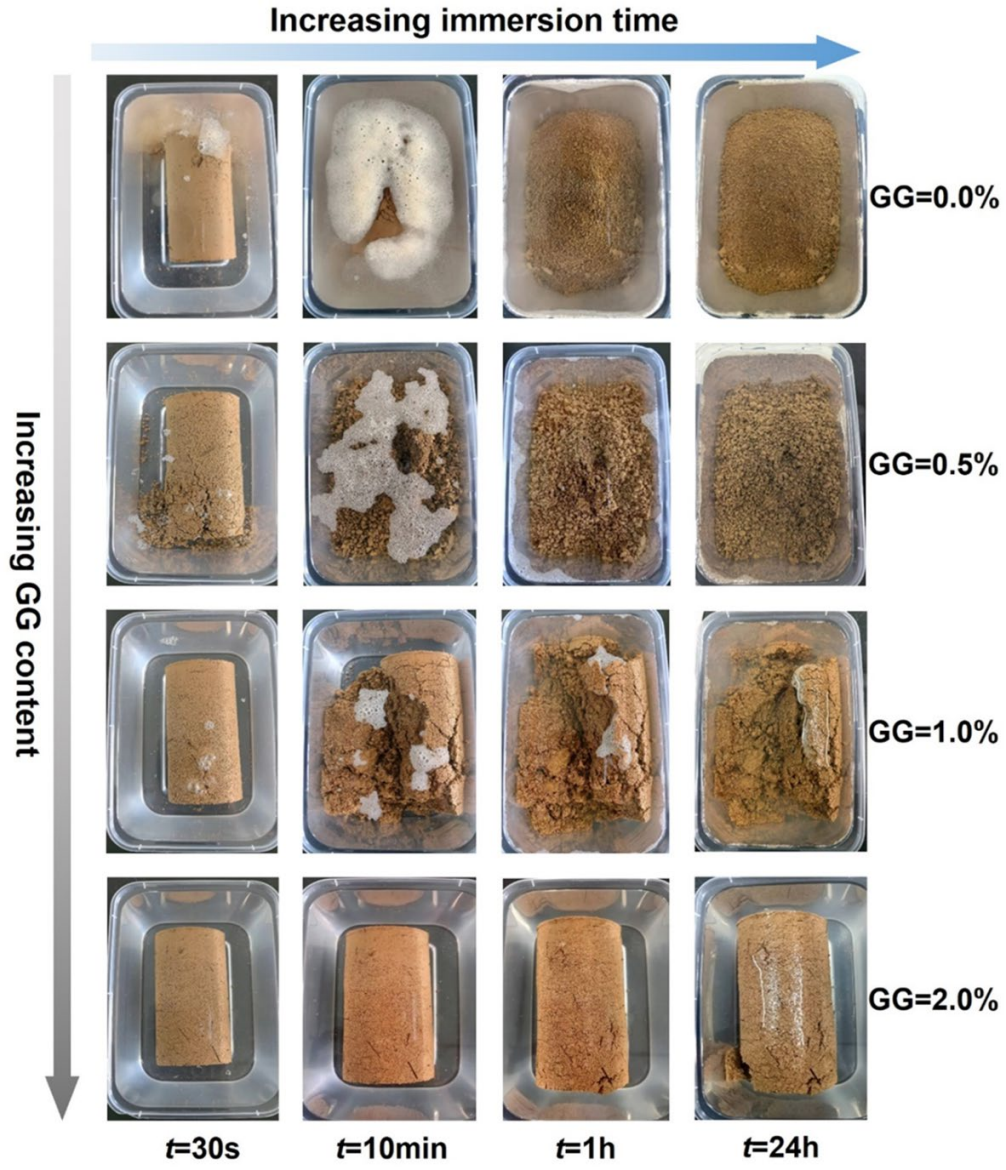
Immersion test results of GG–fiber stabilized loess.

Figure 4 illustrates the immersion test results of loess and GG–fiber stabilized loess specimens at different times. When immersed in water for 30 s, cracks appeared at one end of the loess specimen and discharged a large number of bubbles, with obvious fragments produced; after 10 min of immersion, the specimen disintegrated severely, the water became cloudy, and only the lower end of the specimen remained cylindrical; when immersed for 1 h, the specimen disintegrated completely. For the 0.5% guar gum stabilized loess, the lower end of the specimen cracks and small spherical fragments fall off when immersed in water for 30 s; After 10 min of immersion, the specimen basically completed disintegration, releasing a large number of bubbles floating on the water surface. The results indicate that the water stability of 0.5% guar gum stabilized loess is inferior to that of untreated loess. This is because when the content of guar gum is low, its distribution in the soil is nonuniform. The hydration reaction forms a hydrogel to closely connect part soil particles, causing the pores of the other part of the loess to become larger, and further reducing the original water stability. With the increase of GG content, the water stability of stabilized loess is significantly improved. When the stabilized loess (GG = 2.0%) is immersed in water for 30 s, there is no obvious change in the specimen; cracks appear at the lower end of the specimen and a small portion of particles detach with increasing time, but the overall integrity is remarkably enhanced compared to other specimens. The results indicate that increasing the content of biopolymer can improve the water stability characteristics and effectively reduce the damage caused by hydraulic erosion to the specimen^26,38^.

### Shear strength

**Fig. 5:**
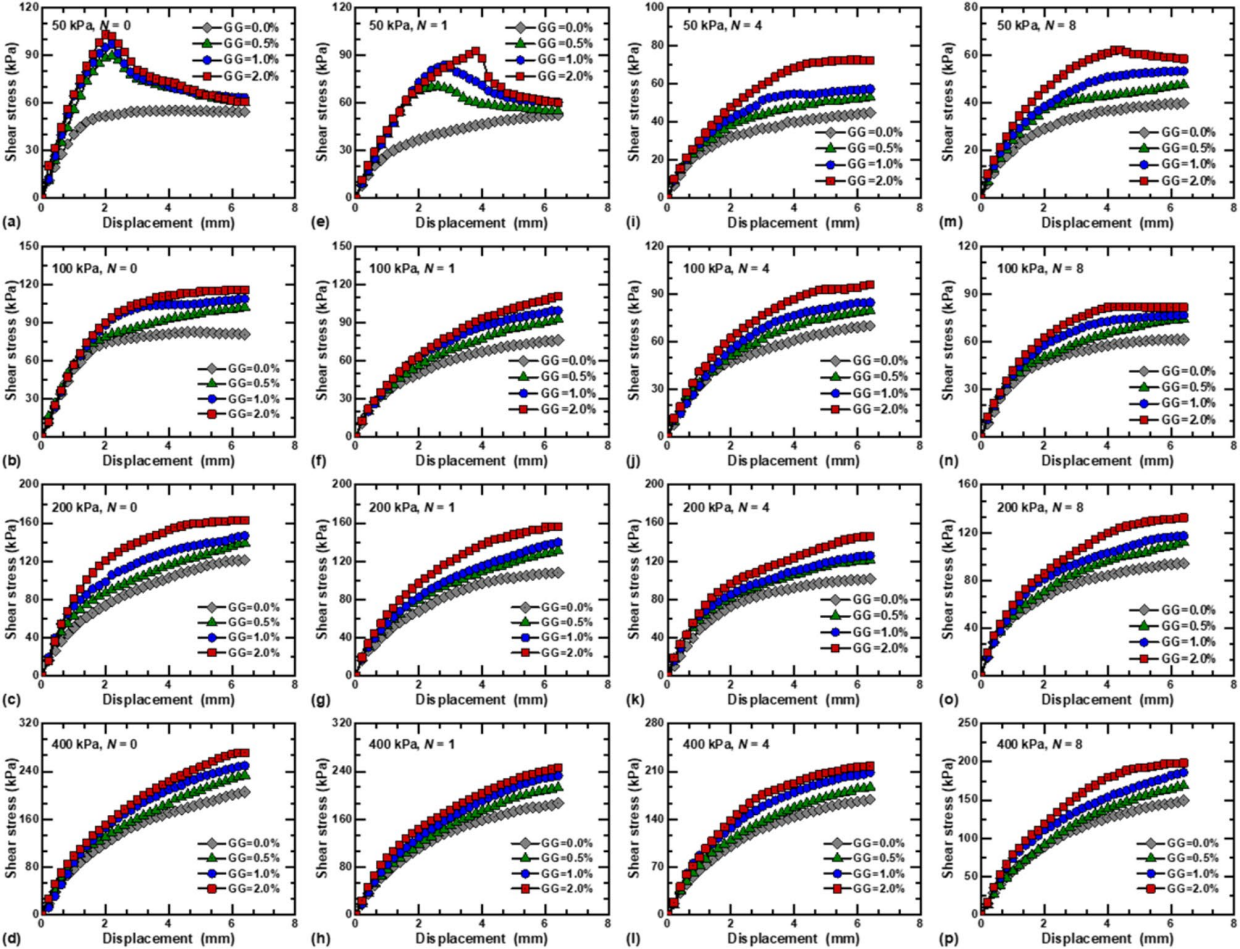
Shear stress-displacement behavior of GG–fiber stabilized loess under different vertical stresses: (**a**–**d**) *N* = 0; (**e**–**h**) *N* = 1; (**i**–**l**) *N* = 4; (**m**–**p**) *N* = 8.

The shear stress–displacement curves for stabilized loess under different GG contents are presented in Fig. 5. Under the vertical stress of 50 kPa, the shear stress of loess stabilized with guar gum increases first and then decreases with increasing displacement, with an obvious peak value, and the stress–strain curves exhibit a strain-softening behavior, as shown in Fig. 5a and e. However, the stress–strain curves of fiber-reinforced loess exhibit strain-hardening under dry–wet cycles. As the vertical stress increases, the stress–strain curves of stabilized loess gradually transition towards strain-hardening, showing two stages of rapid and slow growth throughout the shearing process. This is due to the fact that at higher vertical pressures, the pores are compressed, the contact area between the particles increases and the specimen becomes denser. The compaction effect makes the internal structure of the soil more stable, and the interaction force between the particles is enhanced. When GG = 2.0% and *N* = 0, the peak shear stress increased by 12.43%, 58.28%, and 163.81% under vertical stress of 100, 200, and 400 kPa, respectively, compared with 50 kPa, as shown in Fig. 5a–d. The shear stress of GG–fiber stabilized loess under different vertical stresses is obviously higher than that of specimens without guar gum, and the higher the content of GG, the stronger the shear resistance of specimens. Taking *N* = 0, 400 kPa as an example (Fig. 5d), the treatment of guar gum increased the shear stress from 205.52 kPa (GG = 0.0%) to 233.41 kPa (GG = 0.5%), 249.56 kPa (GG = 1.0%), and 275.43 kPa (GG = 2.0%), which show a notable surge of 13.57%, 21.43%, and 34.01%, respectively. Guar gum can generate a layer of gelatinous film on the surface of soil particles, increasing the cohesion between particles, and forming a guar gum-particles matrix. In addition, guar gum can fill the pores and create a network structure among the particles, which significantly enhances the structural stability of the loess^28,39^.

**Fig. 6:**
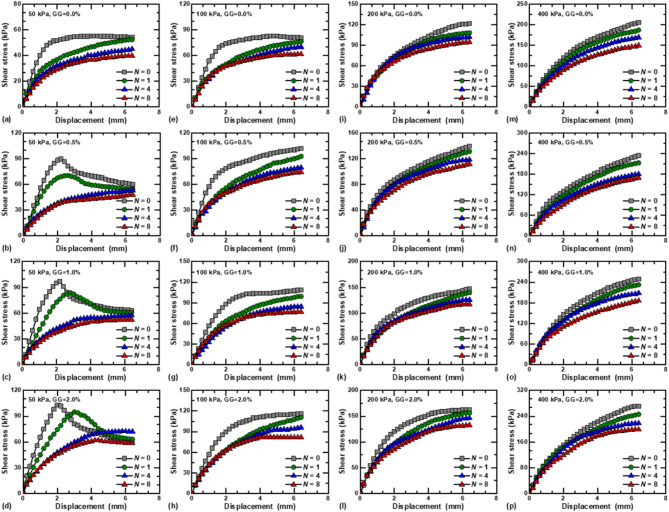
Shear stress-displacement behavior of GG–fiber stabilized loess under different vertical stresses: (**a**–**d**) GG = 0.0%; (**e**–**h**) GG = 0.5%; (**i**–**l**) GG = 1.0%; (**m**–**p**) GG = 2.0%

Figure 6 depicts shear stress–displacement curves of GG–fiber stabilized loess under different dry–wet cycles. Under low vertical stress (50 kPa), the stress–strain curve gradually changes from strain-softening to strain-hardening with increasing *N*, as shown in Fig. 5a–d. However, the different dry–wet cycles processes exhibit strain-hardening types at higher vertical stresses. The dry–wet cycles have a significant degradation effect on both fiber-reinforced and GG–fiber stabilized loess, that is, the peak value of shear stress decreases with increasing dry–wet cycles. For example, when GG = 0.5% and vertical stress is 100 kPa, the peak shear stresses of the specimens are 101.88, 92.48, 79.27, and 73.96 kPa for *N* = 0, 1, 4, and 8, respectively, as shown in Fig. 6f. When GG = 2.0% and vertical stress is 400 kPa (Fig. 6p), The peak shear stress decreased by 9.49%, 19.74%, and 26.93% under dry–wet cycles of 1, 4, and 8, respectively, compared with fiber-reinforced. This is mainly due to the specimen by evaporation and humidification effect of dry shrinkage and wet expansion, the specimen internal micro-cracks continue to expand and evolve, which leads to the gradual reduction of shear strength^3^.

**Fig. 7:**
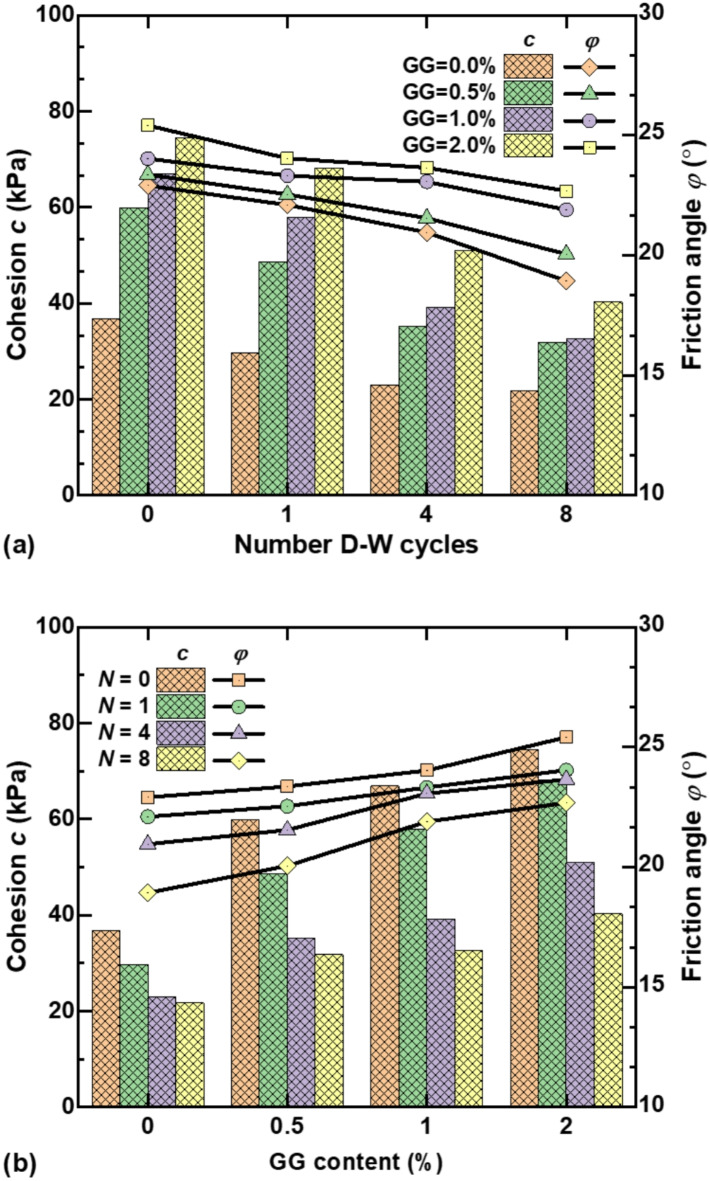
Variation of cohesion and internal friction angle of stabilized loess with: (**a**) dry–wet cycles; (**b**) GG content.

Figure 7 shows the variation of cohesion *c* and internal friction angle $\varphi$ of stabilized loess with GG content and dry–wet cycles. The strength indexes *c* and $\varphi$ of stabilized loess show a decreasing trend with or without guar gum under drying–wetting effects. On the contrary, the cohesion and the internal friction angle increase obviously with increasing GG content under constant dry–wet conditions. When *N* = 0, the cohesion of 0.5%, 1.0%, and 2.0% GG–fiber stabilized loess increased by 62.63%, 81.42%, and 101.79%, respectively, compared with fiber-reinforced loess. When *N* = 8, the cohesion of fiber-reinforced and 0.5%, 1.0%, and 2.0% GG–fiber stabilized loess decreased by 40.85%, 46.98%, 51.25%, and 45.90%, respectively, indicating that dry–wet cycles have a significant impact on the deterioration of cohesive of stabilized loess. From another perspective, compared with fiber-reinforced loess, the friction angle of 0.5%, 1.0%, and 2.0% GG–fiber stabilized loess increased by 1.96%, 4.89%, and 10.91% when *N* = 0, respectively. After 8 dry–wet cycles, $\varphi$ of fiber-reinforced and 0.5%, 1.0%, and 2.0% GG–fiber stabilized loess decreased by 17.37%, 14.17%, 8.86%, and 10.74%, respectively. Compared with cohesion, dry–wet cycles have less effect on the deterioration of internal friction angle, which is consistent with the results of Ma et al.^3^.

**Fig. 8:**
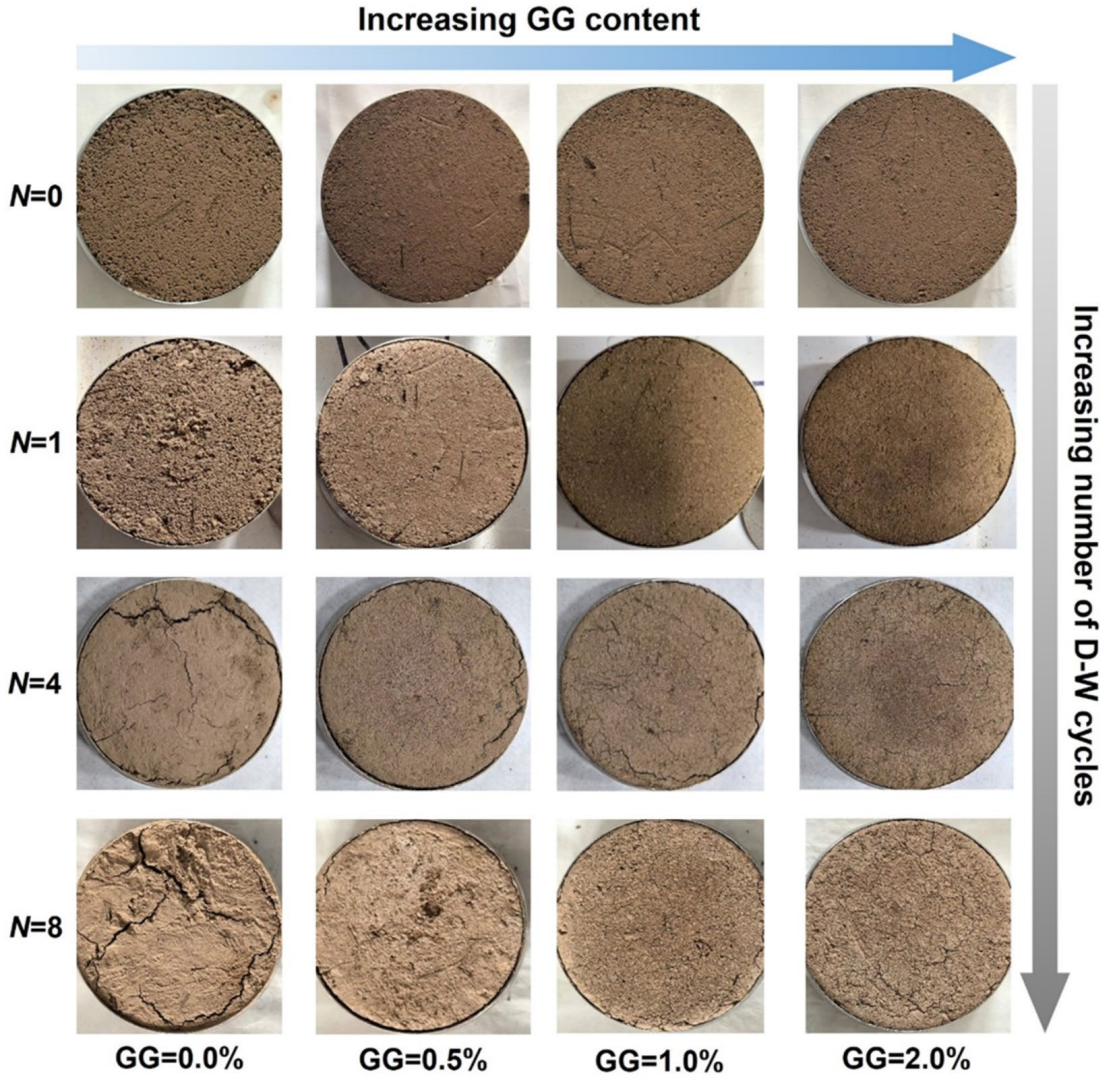
Typical crack morphology on the surface of stabilized loess specimens.

The typical final crack morphology on the surface of stabilized loess specimens with different GG contents and dry–wet cycles is shown in Fig. 8. The drying–wetting effects lead to loose soil structure and weakened adhesion between particles. The loess without guar gum treatment has a fast humidification rate, the volume changes significantly after multiple dry–wet cycles, and a higher degree of crack development, resulting in serious damage to soil structure. For specimens with GG addition, the rate of humidification is slower. Guar gum forms a stable network structure among soil particles, which enhances the cohesion and crack resistance of loess. The stabilized loess with 0.5% GG also shows obvious volume changes, accompanied by the formation of cracks, but the length and width of the cracks decrease. Further increase of GG content can effectively inhibit cracking. For 2.0% GG–fiber stabilized loess, there was no significant change in specimen volume and few cracks occurred on the surface after 8 dry–wet cycles, demonstrating better structural stability. The crack morphology also confirms the correctness of the shear strength degradation results of GG–fiber stabilized loess.

### Water-retention behavior

**Fig. 9:**
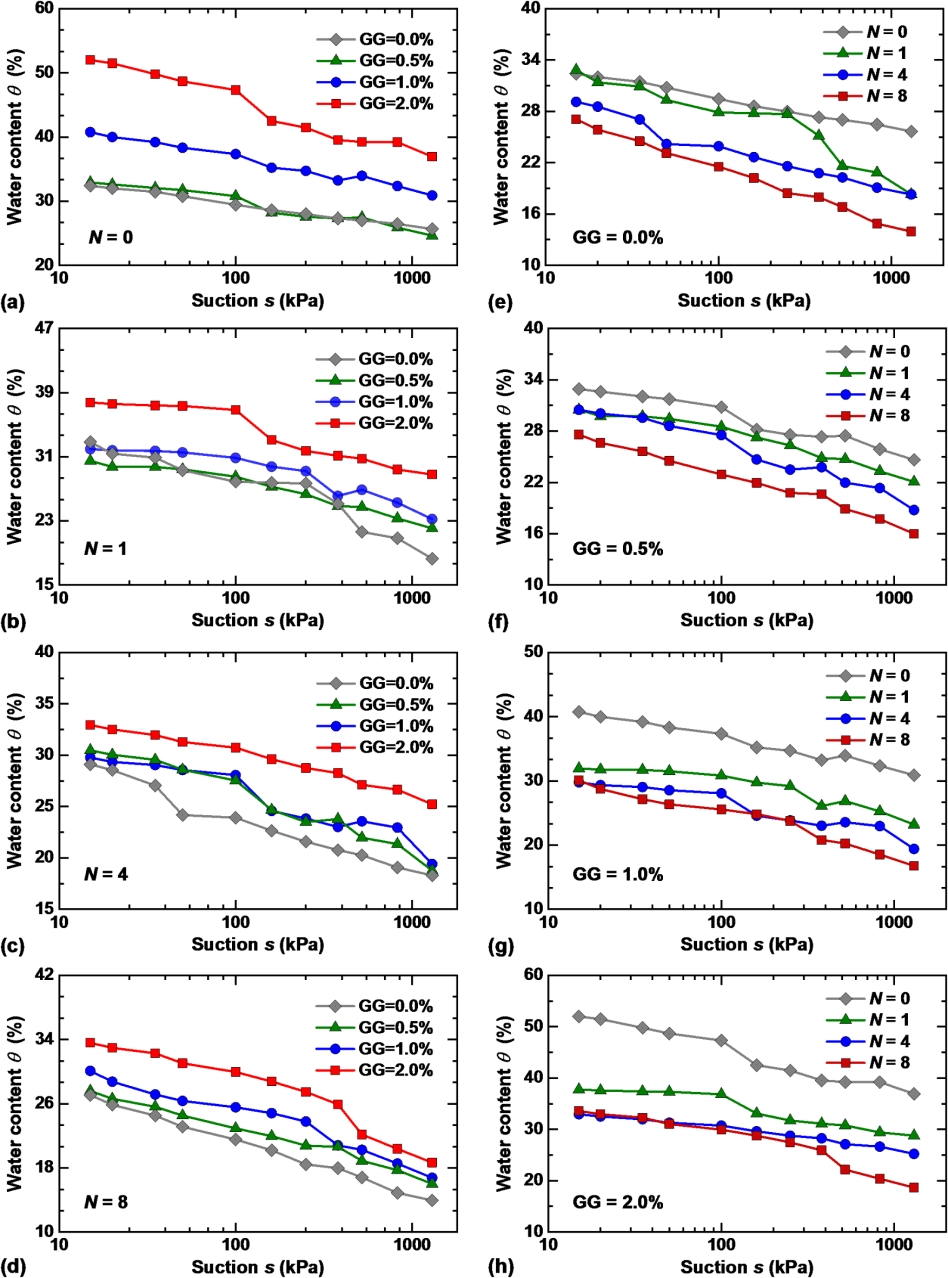
Water-retention behavior of GG–fiber stabilized loess: (**a**–**d**) effect of GG content; (**e**–**h**) effect of dry–wet cycles.

Based on the suction results measured by the pressure plate device, as well as the changes in specimen mass and volume, obtain the soil water characteristic curves (SWCCs) of GG–fiber stabilized loess represented by the volumetric water content ($\theta$)-suction (*s*) relationship, as presented in Fig. 9. Within the measurement range, the trends of the SWCCs of different specimens are basically the same, i.e., the water content of the stabilized loess shows a steady downward trend with increasing suction. Due to its strong water absorption and retention properties, the addition of guar gum to soil can reduce porosity and increase water content. As the GG content increases, the water content of the stabilized loess shows an upward trend under constant *N* conditions, and the water-retention capacity of the stabilized loess is the highest with a 2% GG content. When *N* = 0, the saturated water content of stabilized loess at 2% guar gum is 17.43% higher than that of the specimen without guar gum, as shown in Fig. 9a. It can be observed that when *N* = 1 and 4, the water content of stabilized loess with different GG contents decreased significantly compared with that of untreated specimens (*N* = 0), as illustrated in Fig. 9b and c. However, after 8 dry–wet cycles, the soil structure was gradually stabilized and the differences in water content gradually decreased (Fig. 9d). The SWCCs of GG–fiber stabilized loess under different dry–wet cycles (*N* = 0, 1, 4 and 8) are presented in Fig. 9e–h. During the humidification process, water permeates into the specimen, resulting in the weakening of the bonding of the particles to particles and particles to hydrogels. During the dehydration process, water in the specimen evaporates, thus reducing the water-retention capacity with enlarged pores. The interaction between particles and particles is more sensitive to the drying–wetting effects than that between particles and hydrogels, which exhibits that GG–fiber stabilized loess has stronger water-retention capacity. As shown in Fig. 9h, the saturated water content of stabilized loess with GG content of 0.0%, 0.5%, 1.0%, and 2.0% decreased by 15.87%, 12.45%, 2.13%, and 2.49%, respectively, and the water content at *s* = 1300 kPa is reduced by 18.27%, 14.14%, 8.64%, and 11.7%, respectively, after 8 dry–wet cycles.

### Microscale analysis

**Fig. 10:**
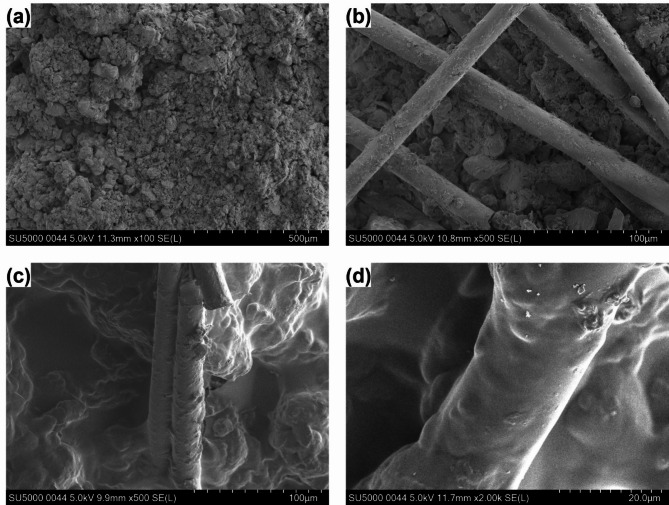
SEM images of (**a**) untreated loess; (**b**) fiber reinforced loess; (**c**,**d**) 2.0% GG–fiber stabilized loess.

Figure 10 shows the microstructure images of untreated loess and 2.0% GG–fiber stabilized loess without dry–wet cycles (*N* = 0). Untreated loess has large porosity, and the connection between soil particles is mostly point-to-point contact, this internal structure determines that loess has poor erosion and shear resistance (Fig. 10a). Fibers are in close contact with soil particles. A single fiber can restrict the relative sliding of soil particles through bending, embedding, and bridging^40^, and multiple fibers can form mesh support, as shown in Fig. 10b. Meanwhile, there is strong friction between the fiber and the soil, which delays the expansion of the internal micro-cracks and enhances the strength and toughness of the soil^2,30,32^. For guar gum, there is an obvious difference with fiber reinforcement, and its stability of loess mainly depends on hydrogel adhesion and pore filling (Fig. 10c,d). When guar gum and fibers are added to loess simultaneously, in addition to their own functions, they also produce a mutual promotion effect. Hydrogel can enhance the interfacial force between particles and fibers, and fibers provide more powerful space conditions for the adhesion of hydrogel-aggregates, forming a more stable “fiber-hydrogel-particles” system^3,30^. Therefore, the strength and water-retention characteristics of stabilized loess are significantly improved under the synergistic of the hydrogel effect of guar gum, the reinforcement effect of fiber, and their interaction.

## Prediction model of SWCC for GG–fiber stabilized loess

For the prediction of SWCCs, there are various models including Brooks and Corey (B–C) model, Van Genuchten (VG) model and Fredlund–Xing (F–X) model^41,42^. Compared to other SWCC models, the V-G model performs better under low suction conditions and effectively captures the water-retention behavior of soils between the saturated and dry ranges. To quantitatively evaluate the SWCCs of GG–fiber stabilized loess, the VG model is applied to fit the measured data ($\theta - s$) obtained from the experiment. Further, a prediction model for stabilized loess that can simultaneously describe GG content and drying–wetting effects is established. The expression of the VG model can be written as^43^:

1 $$\theta \left( s \right) = \theta_{r} + \frac{{\theta_{s} - \theta_{r} }}{{\left[ {1 + \left( {\alpha s} \right)^{n} } \right]^{{\left( {1 - 1/n} \right)}} }}$$

where $\theta_{s}$ and $\theta_{r}$ are the saturated and residual water content, respectively; $\alpha$ and *n* are the soil property parameter.

**Fig. 11:**
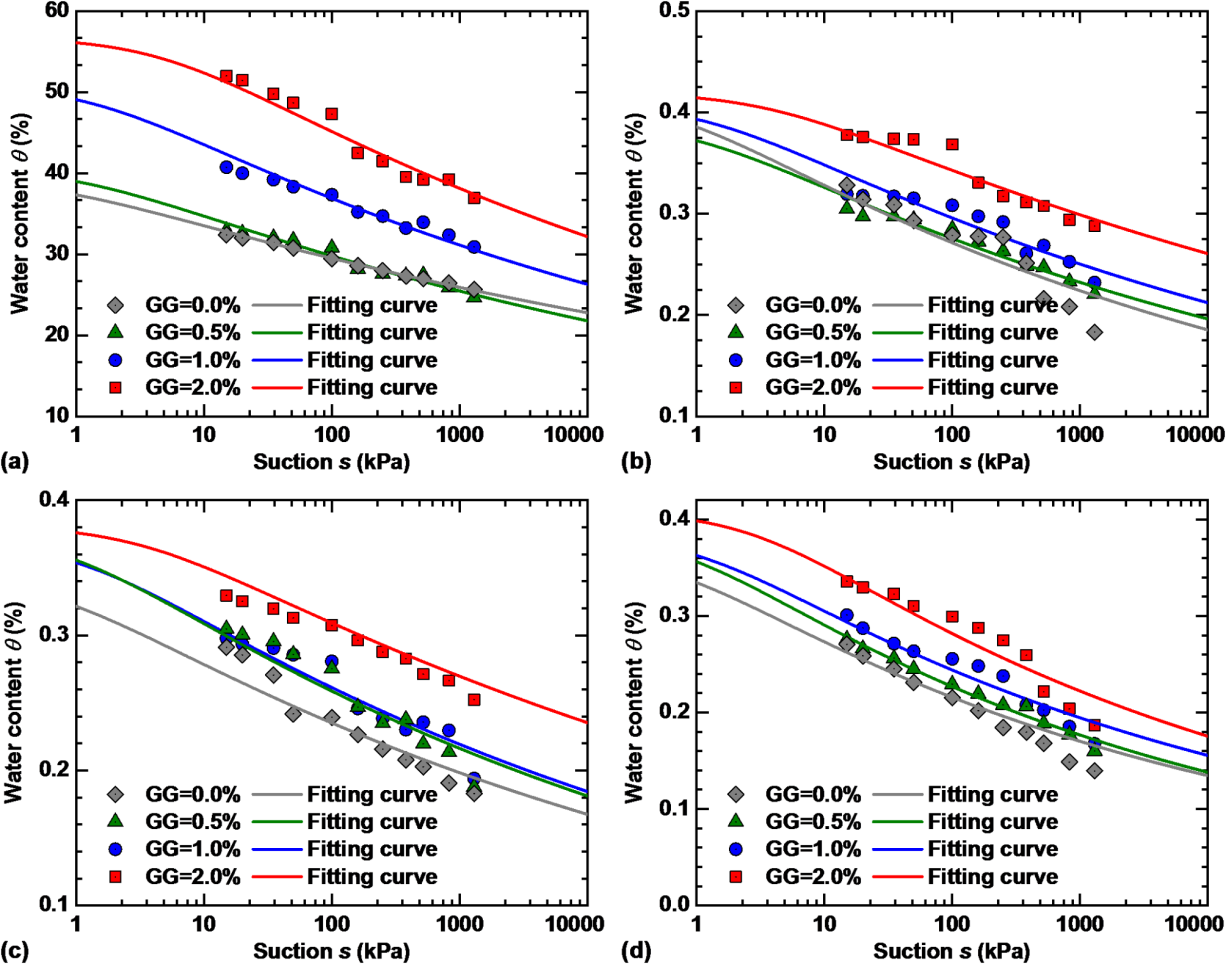
VG model predicted SWCCs of stabilized loess: (**a**) *N* = 0; (**b**) *N* = 1; (**c**) *N* = 4; (**d**) *N* = 8.

According to Eq. (1), the SWCCs of stabilized loess specimens under different dry–wet cycles and GG contents are best fitted, as shown in Fig. 11. The VG model parameters of specimens are summarized in Table 2. Comparison with the measured data indicates that the VG model can adequately reflect the effects of *N* and GG content on the SWCCs of loess, with *R*^2^ > 0.92 of 16 specimens. It can be observed from Table 2 that parameter *n*, which is related to the air entry suction, ranges from 1.06 to 1.11, is independent of dry–wet cycles and GG content. However, parameter $\alpha$ showed obvious regularity with dry–wet cycles and GG content, which decreases with increasing GG content and increases with an increase in *N*.

**Table 2:** VG model parameters of stabilized loess.

Dry–wet cycles	GG content (%)	$\theta_{s}$ (%)	$\theta_{r}$ (%)	*α* (kP^−1^)	*n*	*R* ^2^
*N* = 0	0.0	39.33	1.09	1.57	1.06	0.99
	0.5	40.82	1.24	0.94	1.07	0.98
	1.0	51.00	1.67	0.74	1.08	0.97
	2.0	56.76	1.13	0.21	1.08	0.97
*N* = 1	0.0	41.61	1.15	1.58	1.09	0.92
	0.5	39.13	1.55	1.04	1.08	0.97
	1.0	40.96	1.49	0.82	1.08	0.94
	2.0	41.98	1.01	0.28	1.06	0.94
*N* = 4	0.0	34.45	1.02	1.62	1.08	0.94
	0.5	37.64	1.04	1.16	1.08	0.95
	1.0	36.98	1.26	0.88	1.10	0.95
	2.0	37.14	1.92	0.31	1.06	0.99
*N* = 8	0.0	36.84	0.90	1.65	1.11	0.99
	0.5	38.69	0.98	1.25	1.11	0.99
	1.0	38.55	1.16	0.95	1.10	0.96
	2.0	40.89	0.90	0.34	1.11	0.93

**Fig. 12:**
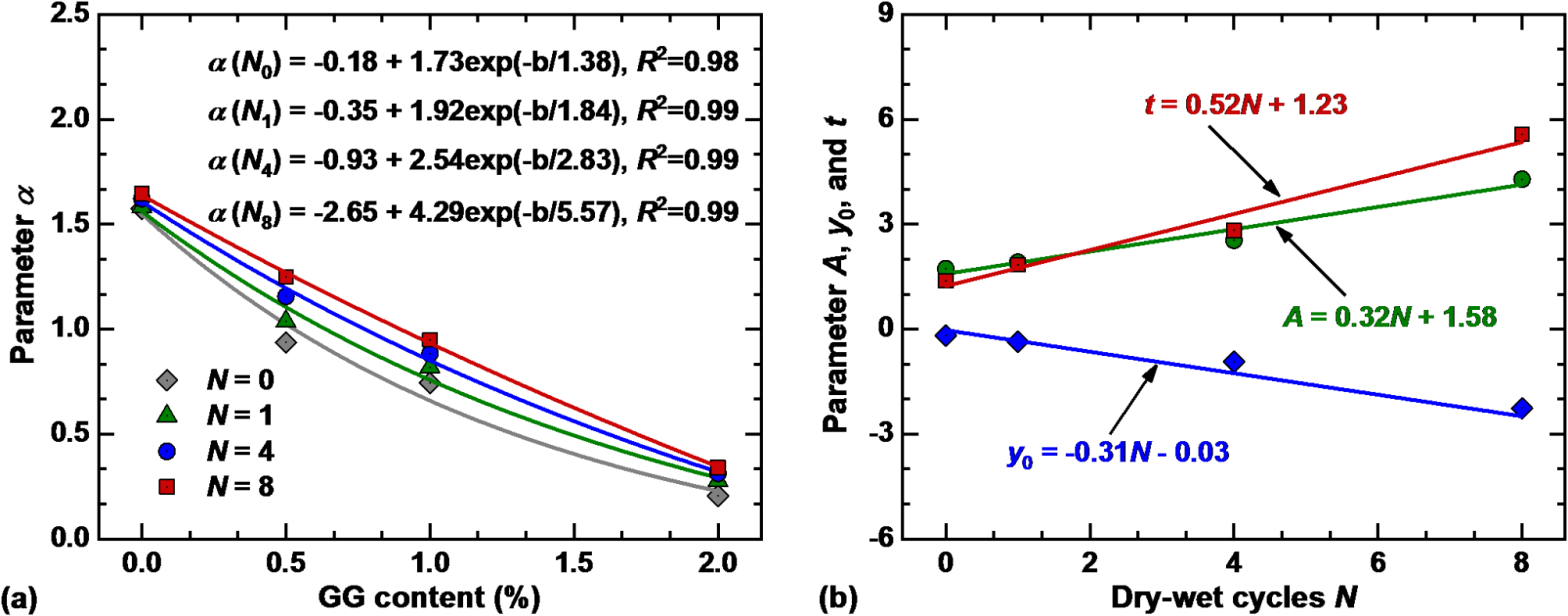
(**a**) relationship between $\alpha$ and GG content; (**b**) relationship between fitting parameters ($y_{0}$, *A*, and *t*) and *N.*

Figure 12a shows the relationship between parameter $\alpha$ and GG content under different dry–wet cycles, which can be represented by the following exponential function:

2 $$\alpha = y_{0} + A\exp \left( { - b/t} \right)$$

where $y_{0}$, $A$ and $t$ are the fitting parameters; $b$ denotes the content of GG. From the fitting results in Fig. 12a, parameters $y_{0}$, $A$, and $t$ are affected by *N*, which can be expressed by linear equation, as shown in Fig. 12b.

Therefore, combining Eqs. (1) and (2), and the fitting function in Fig. 12b, the SWCCs prediction model for GG–fiber stabilized loess is obtained as follows:

3 $$\theta \left( s \right) = \theta_{r} + \frac{{\theta_{s} - \theta_{r} }}{{\left[ {1 + \left( {\left( { - 0.31N - 0.03 + \left( {0.32N + 1.58} \right)\exp \left( { - \frac{b}{0.52N + 1.23}} \right)} \right)s} \right)^{n} } \right]^{{\left( {1 - 1/n} \right)}} }}$$

**Fig. 13:**
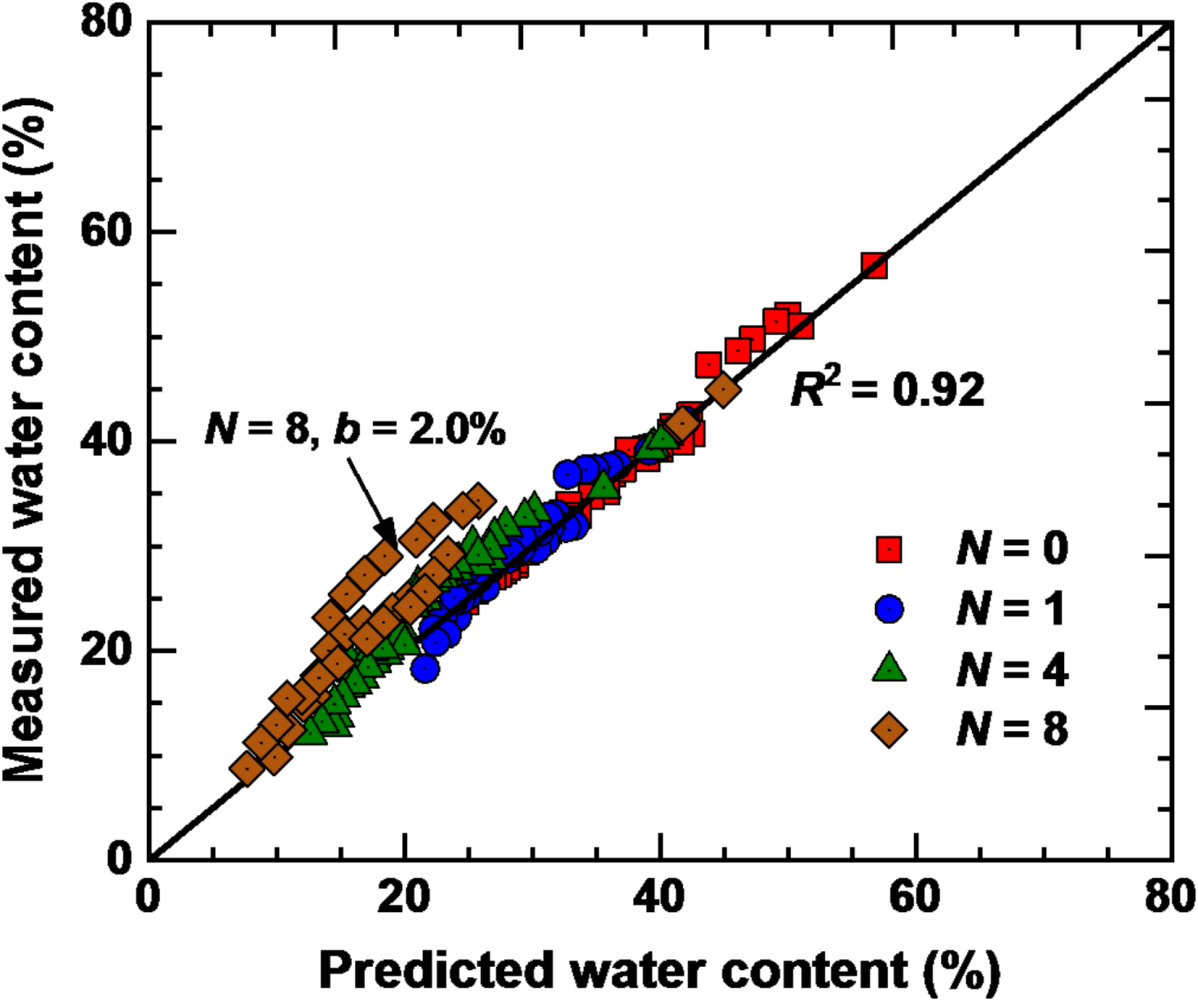
Comparisons between the predicted and measured water content.

The volumetric water content calculated by Eq. (3) incorporates the effect of *N* and GG content. To verify the reliability and accuracy of the model, Eq. (3) is used to predict the test results of SWCCs for 16 specimens. As illustrated in Fig. 13, except for *N* = 8 and *b* = 2.0%, the volume water content calculated by the model is in good agreement with the measured data, with *R*^2^ = 0.92. This indicates that the modified VG model can adequately describe the SWCCs of GG–fiber stabilized loess under different dry–wet cycles with constant fiber content. By analyzing and validating the prediction model, the water-retention behavior of stabilized soil under dry–wet cycles can be better understood, and provide scientific support for the rational use of land and the protection of the ecological environment.

## Conclusions

In this study, a series of laboratory tests, including unconfined compressive strength, immersion, direct shear, soil–water characteristic curve, and scanning electron microscopy test, are conducted to explore the strength and water-retention behavior of GG–fiber stabilized loess under dry–wet cycles. The following conclusions can be drawn:

1. The addition of basalt fiber and guar gum can enhance the compressive strength of loess, and the optimal fiber content is 0.6%. The water stability of loess is poor, and it will completely disintegrate within a short time after immersion. The disintegration resistance of stabilized loess is remarkably enhanced with increasing guar gum content.
2. The shear stress of GG–fiber stabilized loess under different vertical stresses is obviously higher than that of specimens without guar gum, and the higher the guar gum content, the stronger the shear resistance of specimens. The wet–dry cycles have a significant degradation effect on untreated and GG–fiber stabilized loess. Compared with cohesion, dry–wet cycles have less effect on the deterioration of internal friction angle. After 8 dry–wet cycles, the cohesion and internal friction angle of the specimen containing 2.0% guar gum decreased by 45.90% and 10.74%, respectively.
3. Guar gum can generate a layer of gelatinous film on the surface of soil particles, increasing the cohesion between particles and filling pores. In addition, the hydrogel generated by the hydration reaction of guar gum can enhance the interfacial force between particles and fibers, and fibers provide more powerful space conditions for the adhesion of hydrogel-aggregates, forming a more stable “fiber-hydrogel-particles” system.
4. As the guar gum content increases, the water content of the stabilized loess shows an upward trend, indicating a stronger water-retention capacity. In contrast, the drying–wetting effects has a significant deterioration effect on the water-retention capacity of stabilized loess. Furthermore, the SWCCs prediction model of GG–fiber stabilized loess is established by comprehensively considering the influence of dry–wet cycles and GG content based on the VG model, and the prediction results are basically consistent with the measured data.

## Data Availability

The datasets used and/or analyzed during the current study available from the corresponding author on reasonable request.
